# Probing GPCR Dimerization Using Peptides

**DOI:** 10.3389/fendo.2022.843770

**Published:** 2022-07-14

**Authors:** Zara Farooq, Lesley A. Howell, Peter J. McCormick

**Affiliations:** ^1^ Centre for Endocrinology, William Harvey Research Institute, Bart’s and The London School of Medicine and Dentistry, Queen Mary University of London, Charterhouse Square, London, United Kingdom; ^2^ Department of Chemistry, School of Physical and Chemical Sciences, Queen Mary University of London, Mile End Road, London, United Kingdom

**Keywords:** GPCR (G protein coupled receptor), oligomer, protein-protein interaction, peptides, therapeutics targets, pharmacology

## Abstract

G protein-coupled receptors (GPCRs) are the largest class of membrane proteins and the most common and extensively studied pharmacological target. Numerous studies over the last decade have confirmed that GPCRs do not only exist and function in their monomeric form but in fact, have the ability to form dimers or higher order oligomers with other GPCRs, as well as other classes of receptors. GPCR oligomers have become increasingly attractive to investigate as they have the ability to modulate the pharmacological responses of the receptors which in turn, could have important functional roles in diseases, such as cancer and several neurological & neuropsychiatric disorders. Despite the growing evidence in the field of GPCR oligomerisation, the lack of structural information, as well as targeting the ‘undruggable’ protein-protein interactions (PPIs) involved in these complexes, has presented difficulties. Outside the field of GPCRs, targeting PPIs has been widely studied, with a variety of techniques being investigated; from small-molecule inhibitors to disrupting peptides. In this review, we will demonstrate several physiologically relevant GPCR dimers and discuss an array of strategies and techniques that can be employed when targeting these complexes, as well as provide ideas for future development.

## Introduction

G protein-coupled receptors (GPCRs) are involved in a wide range of physiological and signalling processes, with around 30% of currently marketed drugs targeting them, highlighting their pharmacological significance (1). The superfamily of GPCRs is divided into six classes based on functional similarity and sequence homology; Class A (Rhodopsin-like receptors), Class B (Secretin family), Class C (Metabotropic Glutamate receptors), Class D (Fungal Mating Pheromone receptors), Class E (Cyclic Adenosine Monophosphate receptors) and Class F (Frizzled and Smoothened receptors) (2, 3). With the majority of marketed therapeutics targeting the Rhodopsin-like family, in this review, this class of GPCRs will be the focus, with the recognition that parallels can be drawn between classes.

The traditional perception of GPCRs existing as monomeric entities has been overridden by significant evidence suggesting that they can also form oligomeric complexes, such as homo- and heterodimers. It has been demonstrated that the formation of oligomers can impact trafficking, signalling, ligand binding and the overall function of the receptor, which has allowed for these high order complexes to be investigated further (4). Studying protein-protein interactions (PPIs) has become an increasing and developing area of interest in the field of drug discovery. Classically, when two or more protein molecules come together as a consequence of a biochemical incidence, interactions such as hydrogen bonding, electrostatic forces or hydrophobic interactions occur between the protein molecules; these interactions are what are defined as PPIs (5). PPIs can be seen as the protagonist in a vast range of biological processes and perform a principal role in cellular systems of living organisms (6). When operating inside a living organism, 80% of proteins are known to function in complexes, revealing a small amount of proteins that could operate alone (7, 8). PPIs possess some essential properties that have previously been highlighted by Phizicky and Fields, including; 1) having the ability to deactivate proteins, 2) create new binding sites for small effector molecules, 3) permit substrate channelling, 4) adapt kinetic characteristics of enzymes, 5) be regularly involved in downstream or upstream signalling and 6) having the ability to react with alternative binding partners thus modifying the specificity of proteins for their substrates (9). Consequently, PPIs are often responsible for the molecular basis of many diseases and as a result, understanding and having the ability to target these protein interaction networks can not only aid in treating diseases with drug development, but also in prevention and diagnosis (10). Cell proliferation, apoptosis, transcription & translation of proteins are a few essential biological processes that are regulated by PPIs (11), and with around 130,000 reported binary PPIs being accountable for the progression of diseases such as cancer and cardiovascular diseases, PPIs are an attractive pharmacological target (12). The modulation of these PPIs involved in such pathologies are being targeted with drugs that have progressed to the clinic, many for oncologic purposes. Such examples include check-point inhibitors PD-1/PD-L1 inhibitors and cyclin-dependent kinase 4 and 6 (CDK4/6) (11). On average, it is thought that individual proteins can have up to five interacting partners, however if acting as hubs, these interacting partners can increase to over a hundred (13). Due to these high levels of interactions, it can prove difficult when predicting how far these connections can be disturbed (14). It is therefore no surprise that attempting to untangle this complicated web of PPI networks is a major challenge in drug discovery. Moreover, with interfacial areas reaching between ~1500-3000 Å and surfaces being large and flat, targeting these sites can hold many problems (10). As a result, larger drugs and biologics have also been studied to try and overcome some of the problems that small-molecule ligands face when attempting to target these expansive and dynamic surfaces (15). This in itself comes with its own set of challenges, such as biologics not being orally available like small-molecule drugs are, making them more difficult to administer and overall more costly. However, over the last 20 years, research has made it increasingly possible to target such surfaces, with many of these targets being small molecules. It is thought that drugs inhibiting PPIs are expected to be selective due to the fact that their interfaces are likely to be less conserved than that of active sites on protein surfaces (16). Furthermore, individual PPIs have become validated as drug targets with unique precision due to the developments in gene-editing methods (17), allowing for PPIs to be considered as druggable entities. In this review we will discuss PPIs in the context of GPCR oligomerisation, and the different ways to target these interactions, with a focus on the use of peptides as drugs, as well as considering how current methods can be improved and what the future holds.

### Oligomerisation of GPCRs

Oligomeric complexes can be commonly studied as homodimers (referring to two of the same protein molecules forming a complex) and heterodimers (referring to two differing protein molecules forming a complex). The dimerization of GPCRs has become an emerging topic in recent years due to developing evidence suggesting that they do not solely function as monomeric entities (18). The increasing interest in GPCR oligomerisation has led to the prevalence of high-resolution structural information, facilitating the drug development process (18). Techniques such as; Bioluminescence Energy Transfer (BRET), Fluorescence Resonance Energy Transfer (FRET), Proximity Ligation Assays (PLAs), Homogenous Time-Resolved Fluorescence Energy Transfer (HTRF), atomic microscopy and molecular modelling have assisted these findings, in particular with GPCR-GPCR interactions (19–21). As well as homodimerization, GPCRs have the ability to form complexes with different classes of receptors, such as the receptor tyrosine-protein kinase family (21). Heterodimeric complexes possess pharmacological functions that differ from that of homodimers, resulting in unique characteristics, allowing them to be an emerging pharmacological target for a number of disease states (22).

### Techniques Targeting PPIs

In order to aid the design of small-molecule inhibitors that target PPIs, specific parts of the protein, named ‘energetic hotspots’, can be identified. X-ray crystallography, cryo-electron microscopy (cryo-EM) and alanine scanning have been successfully used to identify these areas of interest within specific proteins (23). Other methods used for the identification of PPI inhibitors include the use of fragment-based drug discovery, particularly for the discovery of small-molecule inhibitors, and computational techniques, which are at the forefront of studying PPIs (24). Since the discovery of various GPCR heterodimers and their implication in pathophysiological processes, targeting these PPIs holds great clinical significance; they have slowly gone from being ‘undruggable’, to being considered and appreciated as attractive drug targets (25). Targeting PPIs can usually be approached through the use of small molecules, proteins or peptides (14). Each of these methods is accompanied with benefits and drawbacks due to the level of complexity that is associated with targeting PPIs. However, emerging evidence regarding peptides as disrupting tools has proven to be a successful and preferred approach when targeting GPCR PPIs (14).

#### Peptides – The Perfect Middle Ground?

**Figure 1:**
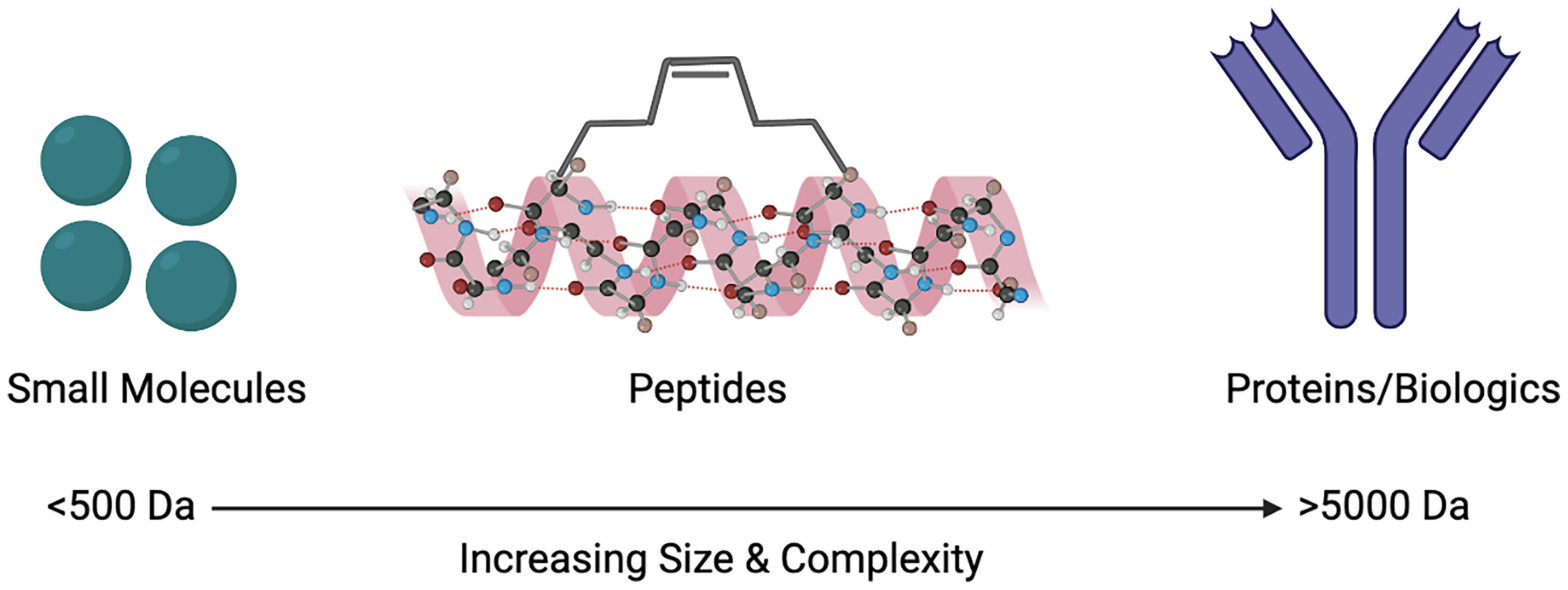
Size comparison of small molecules, peptides and proteins/biologics as therapeutic agents to target protein-protein interactions (PPIs). Created with BioRender.com.

Despite the limitations that peptides as drugs possess, the use of co-crystalised structures have resulted in peptide-based inhibitors (26, 27). These developments have taken into consideration that peptides targeting PPIs are large enough to inhibit the interactions and bind to necessary sites on the protein, as well as having the ability to imitate endogenous interactions (14, 28). Other advances in technology and innovation have also surfaced such as the progression of cell-penetrating peptides (CPPs). Further innovations such as the introduction of unnatural amino acids, the use of macrocycles, as well as stapled and disrupting peptides, all have the ability to improve the oral bioavailability and overall pharmacokinetics of peptides (28). An important feature of GPCRs is their ability to exist in oligomeric complexes, including that of homo- or heteromers. This has sparked an increasing interest in peptides as drugs to target such complexes. Structural information from GPCRs, such as the β1 adrenergic receptor, μ- and κ-opioid receptors and rhodopsin receptors demonstrate that specific regions, namely transmembrane 1 & 2 and helix 8 are commonly conserved interfaces shown in their dimeric forms (29). Within class A GPCRs, dimeric complexes and their interfaces can be understood through the use of synthetic peptides (30, 31). Biased peptide ligands that can selectively target β-arrestin or G protein downstream signalling pathways are also being developed in order to reduce the likelihood of off-target side effects, as well as preventing potential receptor internalisation (32). Although naturally occurring peptides contain 50 or fewer amino acid residues, the FDA defines drugs that are peptides as comprising of 40 or fewer residues, in order to increase the likelihood of successful peptides as therapeutics (33). Here we will discuss some of the different ways that peptides can be used to target PPIs, particularly with GPCRs, and how their size can be seen as the ‘perfect middle ground’ (**Figure 1**).

#### Cell-Penetrating Peptides

CPPs contain 30 or fewer amino acids and have been used in preclinical and basic research over the last 30 years for therapy and diagnosis of several diseases (34). Through energy-independent pathways, these shorter peptides are able to penetrate the cell membrane and transport macromolecules of a hydrophilic nature to the cells without being internalised by endocytosis (35, 36). CPPs are unique as not only do they allow for CPP/cargo complexes to be transported across the plasma membrane, they are also capable of translocating proteins, viruses, macromolecular drugs, imaging agents and nucleic acids across the cell membrane (37). The safety, efficiency and ability to prevent disturbances to the structure of the membrane are crucial advantages of CPPs. However, there is currently a lack of CPP/cargo complexes or CPPs in clinic (34). This is due to; 1) cytotoxicity concerns because of numerous cell types having the ability to internalise CPPs (38), 2) rapid clearance which may prevent the drug being delivered to the desired site (39), 3) possibility of unwanted immune responses leading to undesired side effects, 4) reduced effectiveness of the drug (40) and 5) low specificity. As a result, the need to develop CPPs that are able to be specific, efficient and retain lower levels of toxicity to cells, are required for this species of peptides to be more compatible with clinical applications (34). Pepducins are lipidated CPPs that specifically target the intracellular loops of GPCRs and are a novel approach to modulate the activation of GPCRs (41), as well as improve some of the problems that CPPs as drugs possess. In comparison to their highly conserved transmembrane domains, GPCRs possess more variance in their C-terminal i4 domain and their cytoplasmic loops (il, i2, i3), which are important when it comes to the selectivity, binding and activation of GPCRs (42, 43). The intracellular il-i4 domains of the associated GPCR are used to derive pepducins, and *via* the binding of a lipid group such as a palmitate, pepducins can readily pass through the cell membrane (44). All GPCRs have what is known as an orthosteric binding site; this is where the natural endogenous ligand binds to (45). Additionally, there are other binding sites on GPCRs that are distinct from that of the orthosteric binding site, known as allosteric sites. Ligands that bind to allosteric sites are known as allosteric modulators, and they can either potentiate or inhibit activation of the receptor by its natural ligand or an agonist. Allosteric modulators are important, particularly when specificity in drug design and development can become a problem as they can increase the likelihood of achieving selectivity and reducing adverse side effects. Binding of a topographically distinct site to that of the orthosteric site modifies the receptor conformation and can lead to novel properties and modes of action (46). Intracellular pepducins are thought to bind at allosteric sites on GPCRs, thus acting as allosteric modulators (42), behaving differently to an agonist binding to the orthosteric site, typically located on the extracellular portion of the receptor. In order to activate the receptor at an allosteric site located intracellularly of the receptor G-protein interface, pepducins must penetrate the cell membrane (44). For multiple class A GPCRs, a number of intracellular pepducins agonists have been produced which activate receptors coupling to G-protein subfamilies including Gα_i_, Gα_s_, Gα_q_ and Gα_12/13_ (42). Examples of GPCRs whereby pepducins have been generated include protease-activated receptor 1 and 2 (PAR1, PAR2) (41), melanocortin receptor 4 (MC4) (41), chemokine receptor 4 (CXCR4) (47) and formyl-peptide receptor 2 (FPR2) (48). There has been success with CPP allosteric modulators targeting PPIs involved in cancer, such as the chemotherapeutics paclitaxel, doxorubicin, methotrexate and cyclosporin A (49). With these drugs entering clinic and having positive anti-tumour effects, it provides further hope in the utilisation of allosteric CPPs targeting GPCR PPIs, providing further hope for peptides as drugs.

#### Cyclic Peptides

Cyclisation of peptides has been employed to increase the likelihood of oral administration. Cyclic peptides successfully increase the resistance to proteases, possess a lower binding energy and the structure of the peptide is forced into an active conformation; these allow for the overall affinity and druggability of the peptide to be increased (11). The chemical formation of the macrocycle leads to stability of the compounds and is overall energetically favourable. Nature is to thank for this phenomenon as over the years, macrocyclic peptides have been extracted and isolated from organisms such as fungi, bacteria and plants used to treat a multitude of diseases (50–52). Cyclosporine A, an immunosuppressor, is a prime example of how cyclic peptides can be a administered orally (53). Naturally occurring macrocyclic peptides can be used as chemical scaffolds that have the ability to stabilise peptides that are in linear formations and tend to be formed through a peptide bond formation between the C- and N-terminus, also known as backbone cyclisation (54). Backbone cyclisation has been a successful method of designing peptides as ligands for GPCRs with at least five of this class of peptide currently approved as drugs (32). Pasireotide, octreotide, vapreotide and lanreotide are backbone-cyclic peptides which modulate the somatostatin GPCRs, and bremelanotide targets the MC4 (MC4) (32). Setmelanotide is the most recently approved cyclic peptide for the MC4 receptor used to treat obesity and works by having an agonistic effect (55). As well as obesity, backbone cyclisation has been adapted for therapeutic purposes in diseases such as cancer and human immunodeficiency virus (HIV), whereby molecular grafting techniques have been used in order to allow peptides to selectively bind to their targets by integrating peptide sequences into a number of cyclotides (56). Cyclotides are plant-derived cyclic peptides and have been used as templates in the design and development of peptide ligands for GPCRs (57). They are thought to be more orally bioavailable in comparison to non-cyclic peptides and have a high stability due to their cyclic cystine-knot motif which is comprised of three conserved disulphide bonds, providing rigidity to the structure (58–60). Due to the difficulties surrounding peptide-based drug design for GPCRs, cyclotides have been used as potential grafting scaffolds in order to tackle some of the challenges that peptides as drugs hold (61). Such examples of studies include; targeting the bradykinin receptors to treat chronic pain (62), development of selective and stable agonists of the MC4 receptor for the use of tackling obesity (63) and generating antagonists for the CXCR4 receptor, which is known to be involved in diseases such as cancer and HIV-1 infection (64). As a result, cyclotide templates could have the ability to provide an important way to facilitate the generation of peptide ligands for GPCRs that could overcome some of the problems that linear peptides hold (61).

#### Stapled Peptides

**Figure 2:**
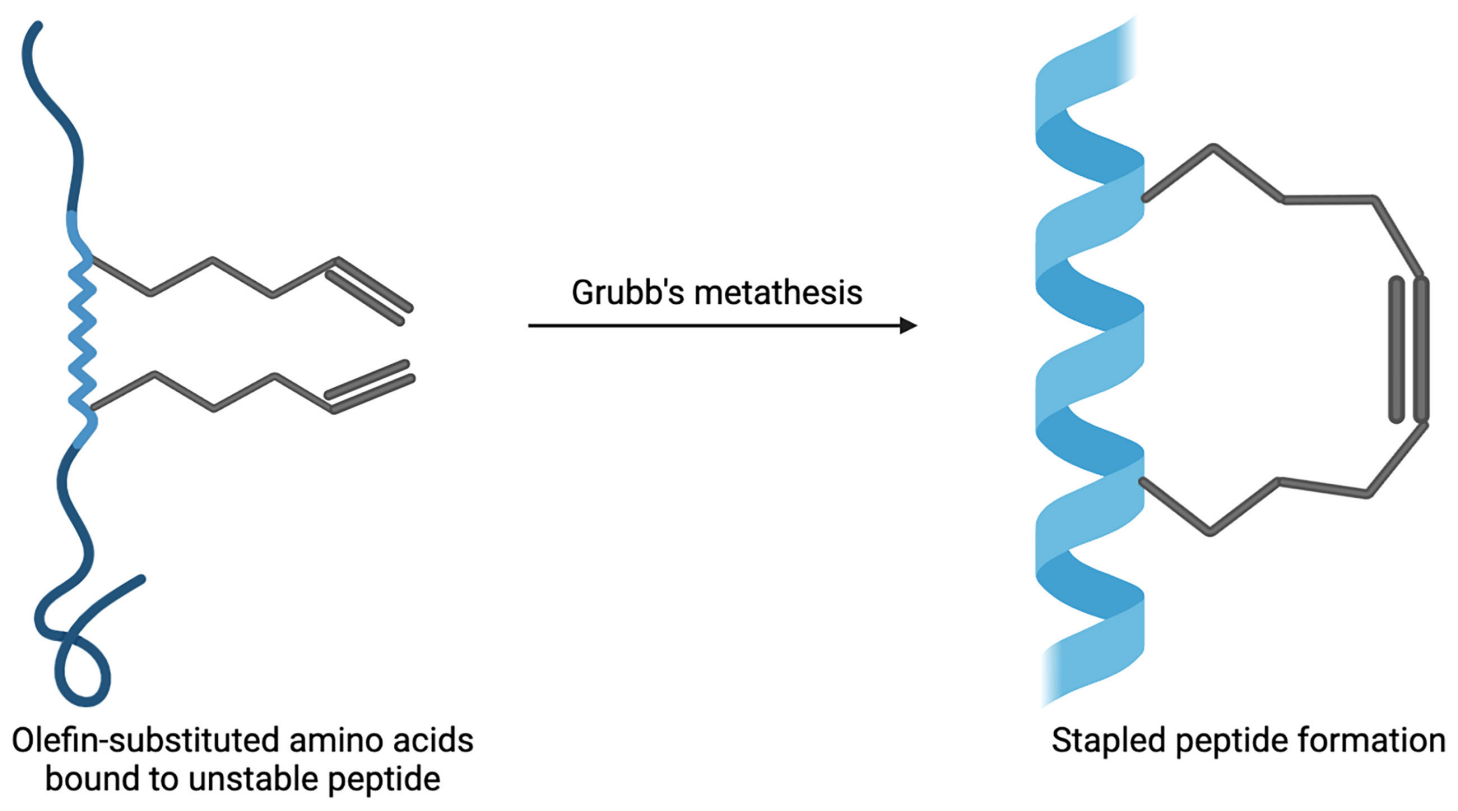
Example of stapled peptide formation. Created with BioRender.com.

Stapled peptides are an evolving technique used to target PPIs that have the ability to overcome some of the problems that large biologics and small-molecule inhibitors have faced. These include not being able to arrive at the intracellular targets, possessing low bioavailability and not being successful in tackling the sizeable and flat nature of the surface of PPIs (65). Hydrocarbon-stapled α-helical peptides are synthesised small proteins secured into their bioactive structure *via* the introduction of a chemical linker that is site-specific and designed to mimic the secondary structure of proteins (24). Stapled peptides can be seen as the best of both worlds when targeting PPIs; they are the middle ground between small molecule inhibitors and biologics. They possess the biophysical properties of small molecule inhibitors, yet can retain the high binding affinity of protein-based inhibitors (11). The improved affinity, resistance to proteolysis and increased likelihood of cell penetration are a few of the reasons as to why stapled peptides are advantageous (66–68). The α-helix is an important structural component of proteins that facilitate intracellular PPIs involved in many biological processes and pathways (69, 70). The enforcement of the α-helical conformation includes the use of non-native amino acids that are positioned on the same helix face of the peptide α-helix which are then ‘stapled’ together *via* covalently bonded sidechains (71, 72). When tackling resistance towards proteases, the use of non-native amino acids being introduced into the peptide sequence can support the backbone of the peptide and overcome difficulties with unstable conformational changes that can occur when the protein has not fully folded (73). The modification of peptides has developed over the years and was initiated by Verdine et al. through the development of the first all-hydrocarbon staple (74). This work lead to the Grubbs catalyst being used to make a cross-link for the first time on a peptide template (65). Another crucial example involves the Bcl-2 family; it has up to four conserved Bcl-2 homology (BH) domains that possess α-helical segments. This was utilised by Walensky who developed hydrocarbon-stapled peptide helices targeting the BH3 domains (75). The production of this stapled peptide demonstrates that they have the ability to be very stable, increase the permeability of cells, as well as be highly resistant to proteolysis (75, 76). The chemical synthesis of stapled peptides uses unnatural amino acids which bear olefin sidechains that are ‘stapled’ to the synthetically produced amino acid domain of interest (**Figure 2**) (24). The use of non-natural amino acids as building blocks means that diverse chemical compounds can be synthesised and have the ability to be modified both functionally and chemically, in order to achieve the desired molecule (77). These reasons make the use of stapled peptides very appealing when targeting PPIs for therapeutic applications. An example using stapled peptides within class A GPCRs is demonstrated through the dimerization between cannabinoid receptor 1 (CB_1_) and serotonin receptor 2A (5HT_2A_) (29). It has been shown that in the presence of Δ-9-tetrahydrocannabinol (THC), unwanted effects of cognitive impairment is induced by the oligomerisation between CB_1_ and 5HT_2A_ (78). In order to disrupt the CB_1_-5HT_2A_ heteromer, a stapled peptide was designed based on the truncated transmembrane domain 5 (TM5) of CB_1_ fused to a cell penetrating HIV-TAT (GRKKRRQRRR) sequence; *in vitro* assays showed that the stapled peptides designed were effective at disrupting these heteromers (29). These developments in research have allowed for further studies and developments to be carried out in order to target otherwise undruggable GPCR heterodimers.

### The Antibody Approach – Does Size Matter?

The development of monoclonal antibodies (mAbs) (14) as targeted drugs are an essential tool for cancer research, with a number of drugs being approved by the FDA (79). Phage display is an incredibly useful molecular diversity technique that studies binding sites of receptors, interactions between proteins and ligands, and is also used to improve the affinity of proteins towards their ligands and other binding partners (80). This technique was used to design the first inhibitory antibody (81) and has been used to design a multitude of high affinity immunoglobulin domains (82). Such biologics have been successfully developed to target PPIs involved in breast cancer. Human epidermal growth factor 2 (HER2) has been used as an important diagnostic tool in the treatment of certain breast cancers that express this protein on the surface of the tumours. Trastuzumab, a monoclonal antibody, has been a efficacious drug used to target the over-expression of the HER2 protein (83, 84). Although there are major advances in the field of targeting PPIs with protein-based compounds, there are still huge limitations with this approach; challenges of mAbs entering cells (14), undesirable side-effects, low oral bioavailability and high cost of development. Nanobodies have been used as a potential alternative to mAbs, which allow for the discovery of more stable and simple compounds that have been found to be successful in targeting GPCRs for not only therapeutic purposes, but also for research and diagonstics (85). Unlike antibodies which have two variable domains, nanobodies have only a single variable domain allowing for more stability and a more straight-forward manufacturing process due to their smaller size (86). However, like mAbs, nanobodies also have drawbacks; these small monomeric entities often induce kidney toxicity due to their rapid renal clearance which in turn limits a high amount at the site of interest (87). The use of mAbs and nanobodies as targeted drugs requires further improvements to allow them to be more desired as therapeutics for PPIs.

### Small Molecules – Do Good Things Come in Small Packages?

**Figure 3:**
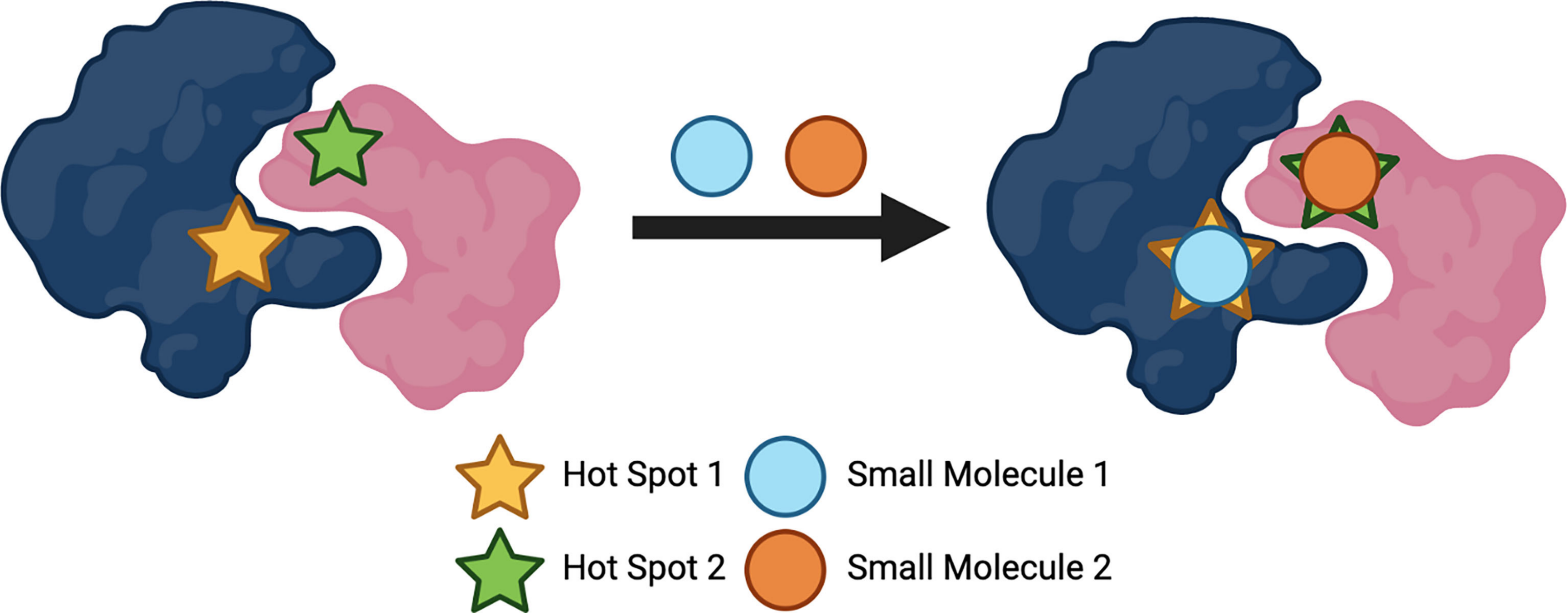
Protein 'hot spots' used to target protein-protein interactions (PPIs). Created with BioRender.com.

Due to the size of the surface of PPIs, designing small molecules that are successful in disrupting these interactions has presented difficulties. Unlike enzymes or receptors whereby stabilisation occurs *via* the active or binding sites, proteins that are hidden within the PPI form large interfaces that can have a magnitude of thousands of Å (2) (88). As well as size being a problem, the many features to consider due to the diversity and flatness of the binding sites within the interfaces of PPIs, presents difficulties when producing effective inhibitors (14). With these factors in mind, designing small molecules could seem superfluous. However, over the years, there has been developments in the number of small-molecule inhibitors targeting PPIs. In 2018, it was reported that 44 published small-molecule inhibitors targeting PPIs exist, with the majority for the indication of oncology (28). Nonetheless, how is it possible to develop small-molecule inhibitors for PPIs? Pockets, indentations and clefts are thought to be embossed on the surfaces of the proteins, suggesting there is appreciably more to consider when describing their ‘flat’ feature (89). It is thought that for a PPI to be disrupted effectively, the whole PPI surface is not required to be interacted with; particular amino acids within the surface can be targeted (90). These so-called ‘hot-spots’ (**Figure 3**) are fascinating entities as they are generally clustered at the centre of the protein interface and tend to be of the size and area that equates to a small molecule; they also have the ability to modify their conformation and possess hydrophobic characteristics (91). These ‘hot-spot’ binding pockets are found to be within a range of 250-900 Å (2) of size, as is found with the majority of the clinical-stage PPI inhibitors (92, 93). Small molecule drugs not only benefit from their overall cost, but are more likely to be orally bioavailable, have a higher metabolic stability, possess a longer shelf-life, as well as having overall advantageous permeability and pharmacokinetics (94, 95). Crucial oncologic PPIs involving members of the Bcl-2 (B-cell lymphoma 2) family of proteins, which regulate apoptosis, are examples of being able to be targeted by small molecules (90). Oncology is the largest field of medicine that benefits from small-molecule PPI inhibitors and will continue to profit from the development of these compounds (28). There is a large number of small molecules that are currently approved to target several GPCRs for a wide variety of disease states, including high blood pressure, type-2 diabetes, pain and schizophrenia; some of these GPCRs include adrenoreceptors, acetylcholine receptors, dopamine receptors, histamine receptors, serotonin receptors and opioid receptors (96). The use of small molecule allosteric modulators that target PPIs has also become an emerging topic of interest within this field of research. As previously mentioned allosteric modulators target sites on proteins that are topographically distinct to that of the orthosteric binding site (46). Allosteric modulators usually have differing and enhanced physiochemical properties that ligands which solely bind to orthosteric binding sites may possess, which could aid in the design and development of ligands which target PPI surfaces. Studies have shown that targeting the heterodimeric gamma-aminobutyric acid B (GABA_B_) receptor, using positive allosteric modulators (PAMs) that can bind at the interface of the transmembrane domains, can control the activation of this class C GPCR dimer (97). PAMs are allosteric modulators that enhance the agonist affinity for the receptor and negative allosteric modulators (NAMs) will inhibit the agonist affinity (98). The GABA_B_ PAMs bind in the transmembrane interface which is conserved across most GPCRs, making these findings important for not only class C GPCRs, but for the whole superfamily (97). Importantly, the discovery of allosteric modulators is mostly limited to computational analysis, providing an obstacle for the development of these compounds. However, recent developments in computational and experimental strategies have allowed for the identification of allosteric sites to become less challenging, which allows for the development of allosteric modulators to become more likely (99, 100). These are crucial findings for targeting GPCR dimers as they provide the possibility in developing allosteric modulators that can alter the activity of oligomers that are involved in diseases, allowing them to be pharmacologically attractive compounds.

### GPCR Dimers as Physiological Targets

With GPCR dimers becoming an attractive target for many diseases, the advancement in technology and demand has allowed for a number of GPCR dimers to be identified and discovered. Some of these physiologically significant oligomeric complexes include; CXCR4 (101), CXCR4 and cannabinoid receptor 2 (CB_2_) (22), CB_2_ and HER2 (21), dopamine receptor 1 (D_1_) and dopamine receptor 2 (D_2_) (102), glutamate receptor 2 (mGlu_2_) and 5HT2A (103), CB1 and 5-HT_2A_ (104) and D_2_ and adenosine receptor 2A (A_2A_) (105). We will discuss the biological relevance of these dimeric complexes as well as explore the different techniques that have been used to target and approach them so far.

#### CXCR4 Receptor Homomer

CXCR4 is one of 19 known chemokine receptors and is activated by its endogenous ligand, stromal cell-derived factor-1 (SDF-1) or CXCL12 (64). CXCR4 has been linked to HIV, as well as many different types of cancers due to its ability to stimulate angiogenesis, tumour growth, metastasis and tumour survival (106, 107), making it an important drug target. Studies have shown that CXCR4 homodimers have been detected in tumour cells, not only in the cytoplasm but also on the surface of the cell membrane (101). Wang et al. concluded that interactions between CXCR4 receptors might have an important part to play in the migration of cells and the homodimeric forms that are found intracellularly as well as on the cell surface, making it a suitable target to manage metastasis of tumours (101). Heterodimerisation of CXCR4 has also been established with the discovery of CXCR4 and chemokine receptor type 2 (CCR2) through the use of BRET (108). The function of oligomeric CXCR4 complexes can differ from that of its monomeric entity as the function of the receptor can change (109, 110). An antibody-based approach has been used to target this receptor using nanobodies; small proteins (12-15 kDa) made from single variable domains of heavy-chain antibodies that have the ability to act as successful conformational sensors (111). The use of nanobodies have the ability to bind to CXCR4 and have various advantages over the use of small molecules, such as possessing longer half-lives and demonstrate higher selectivity (112, 113). The development in this technology to target dimeric complexes of CXCR4 has provided hope with targeting this receptor, and other GPCRs for therapeutic purposes. Through the continued improvement with emerging technologies, as well as growing crystallographic data, targeting GPCR PPIs, such as CXCR4 oligomers through the use of peptides could eliminate the current problems that existing techniques possess.

#### CXCR4-CB_2_ Heteromer

As well as possessing the ability to form homodimers, CXCR4 can also form complexes with other GPCRs. One example is CB_2_, another class A GPCR part of the endocannabinoid system, that is also known to impact tumour growth and metastasis. CB_2_ is primarily expressed in the immune system and the peripheral nervous system (114, 115) and is an attractive pharmacological target for a number of different illnesses, particularly its upregulation in a number of different cancers (116). Inhibition of receptor activation has been observed *via* allosteric interactions within heteromers containing the chemokine receptors (22). The CXCR4-CB_2_ heterodimeric complexes has been found in human prostate and breast cancer cells (22). It is thought that CXCR4 can be silenced when in a heterodimeric complex with CB_2_, which allows for the function of CXCR4 to be inhibited, reducing tumour growth or metastasis (22). Studies have shown that upon agonist activation of CXCR4 and CB_2_ simultaneously, the functions of the malignant tumour cells were reduced (117). At least one biotech company in South Korea, GPCR Therapeutics, whose major focus has been GPCR heteromers, in particular oligomers linked to CXCR4, has recognised the potential for targeting heterodimers in oncology.

#### CB_2_-HER2 Receptor

GPCRs can also form dimeric complexes with other families of receptors; in HER2-positive breast cancers, CB_2_ can form a heteromer with HER2, which belongs to the receptor tyrosine kinase family of receptors (21). HER2-positive breast cancer is a specific subtype of breast tumours and is responsible for approximately 15-20% of malignancies (118). Due to the large number of tumours that present with this subtype, the production of targeted therapies towards HER2-positive tumours has been necessary, and with the successful production of targeted therapies such as Trastuzumab, patient outcome has considerably improved. However, new therapeutics are required for the tumours that are resistant to these targeted therapies, as well as severe cardiac related side effects (119). Evidence suggests that in breast cancer cells, including that of metastatic breast tissues, HER2 physically interacts with CB_2_, forming a heterodimeric complex and is correlated to a poor patient prognosis; this corresponds with the fact that 91% of HER2 positive breast tumours also show CB_2_ expression in immunohistochemical analysis (21, 120). Studies have shown that in HER2 positive breast cancer cells, the use of cannabinoids, such as Δ-9-THC or JWH133, a synthetic CB_2_ specific agonist, can reduce the progression of the disease by a decrease in the tumour number, growth and metastases (120). Trastuzumab is a monoclonal antibody that is used to selectively target HER2 positive breast cancer and has been successful, to a certain extent (83, 84). However, due to approximately 75% of patients not responding to this treatment because of an increase in resistance, other methods to target HER2 positive breast cancers are necessary (120). Novel agents or the use of this mAb in combination with other anti-cancer drugs is a potential option, as well as directly targeting the CB_2_ receptor due to its prevalence in HER2 positive breast cancers.

#### D_1_-D_2_ Receptor Heteromer

Dopamine 1 (D_1_) and dopamine 2 (D_2_) receptors are the two predominant dopaminergic receptors that are found in the part of the brain which is responsible for controlling several aspects of cognition, known as the striatum (121). Dopamine, which is the endogenous ligand for both of these receptors, is a crucial neurotransmitter that controls numerous physiological functions such as emotion, learning and behavioural characterisitcs (121). Abnormal signalling of D_1_ and D_2_ has been linked to a number of neuropsychiatric disorders such as schizophrenia, drug abuse, autism, and Parkinson’s Disease (PD) (122). As a result, the development of ligands that target these receptors in order to treat the variety of neurological related disorders is desired. The dopamine receptors are structurally similar and therefore designing compounds that target each dopamine receptor specifically can become a challenge (123). However, with the occurrence of the D_1_-D_2_ complex, this could allow for drug discovery to be more manageable. Within the brain, D_1_ and D_2_ receptors have been thought to form heterodimeric complexes, which has been established through techniques such as FRET (121). In comparison with the monomeric entities of each receptors themselves, this heteromer engages in an alternative pathway of signal transduction (124). The D_1_-D_2_ heteromer has been shown to have important physiological relevance with diseases such as drug addiction and schizophrenia, which allow for the this complex to be of crucial therapeutic significance (121). Zhuang et al. have recently discovered the first cryo-EM structure of the wild-type D_1_ receptor in complex with the G_s_ protein bound to agonist apomorphine or with selective agonists SKF81297 and SKF83959 (122). This structural information will be crucial for the design and development of drugs selectively targeting not only the D_1_ receptor, but also D_1_ receptor heteromers which will in turn help to treat central nervous system (CNS) disorders. SKF83959 has been found to be a D_1_-D_2_ heteromer specific ligand that has allowed for the isolation of this complex, which has previously been difficult due to dopamine agonists activating both D_1_ and D_2_ homomers as well as this dopamine receptor heterodimer (125). The development of this ligand and the knowledge of its where it binds to, provides useful information and an important stepping stone towards the development of potential disrupting peptides to target this complex.

#### D_2_-A_2A_ Receptor Heteromer

Dopamine receptors have the ability to form heterodimers with other GPCRs that are not within the dopamine family of receptors. In this case, the D_2_ receptor has been shown to form a heterodimeric complex with A_2A_ in the cell membrane (126). The A_2A_ receptor is a useful pharmacological target for novel drug development as it plays a crucial role in regulating blood flow and oxygen consumption required for the heart, as well as the neurotransmitters within the CNS (127). The existence of the D_2_-A_2A_ heterodimer is thought to be involved in striatopallidal γ-aminobutyric acid (GABA) pathways where D_2_ receptor trafficking and signalling is modulated through A_2A_ receptor antagonisation (105). *In vitro* analysis has indicated that the high affinity binding of D_2_ agonists is reduced *via* the activation of A_2A_, which suggests that there are possible allosteric interactions that are influencing the signalling of the D_2_ receptor (128). These allosteric interactions have been associated with the neuroleptic effects of A_2A_ agonists (105), which indicates that this heteromer is an important target for many neurodegenerative related diseases. Moreover, a substantial amount of evidence and experimental findings have demonstrated that the D_2_-A_2A_ heterodimer plays a significant part in the basal ganglia (126), which is linked to PD. In order to target this heterodimeric complex, peptides that can have antagonistic effects towards the D_2_ receptor and agonistic effects for the A_2A_ receptor could represent potential therapeutic importance.

#### CB_1_-5HT_2A_

CB_1_ is a major receptor of the endocannabinoid system, along with CB_2_. It is primarily expressed in the brain and CNS where it modulates neurotransmitters such as dopamine and serotonin (104). 5-HT_2A_ is one of three subtypes of the 5-HT_2_ receptors which are a subfamily of the serotonin (5-HT) receptors (129). CB_1_ and 5-HT_2A_, both class A GPCRs, are thought to form a heterodimeric complex, which has been shown to be expressed in the brain of mice whereby the complex mediates cognitive effects that are elicited by THC (104). Studies carried out have demonstrated that the CB_1_-5HT_2A_ heterodimer can be modulated to achieve separation of the cognitive damage that is stimulated by THC from antinociceptive properties; these are favourable *via* the selective pharmacological blockade or disruption of the PPI by transmembrane interference peptides (78). Botta et al. found that through the use of an aryl-hydrocarbon-stapled CB_1_ TM5-mimicking peptide to target the CB_1_-5HT_2A_ receptor heterodimer, the stapled peptide resulted in an enhancement of proteolytic resistance as well as improved α-helicity, providing confidence for the use of stapled peptides (29). Targeting this heterodimer can have huge advantageous results for cognitive related disorders, with the use of disrupting peptides demonstrating their potential significance and successful use for other GPCR oligomers (104).

#### mGlu2-5HT_2A_ Receptor Homomer

5-HT_2A_ can dimerise with the mGlu_2_ receptor, a class C GPCR. Individually, both receptors have been associated with the disordered physiological processes of neuropsychiatric illnesses such as schizophrenia (103). Affecting around 1% of the population, schizophrenia is a distressing mental disorder that is not fully comprehended, however serotonin, glutamate and dopamine receptors have all demonstrated relevance in the disease (130). There have been a number of studies that have shown the involvement of the 5HT_2A_ receptor with compounds such as lysergic acid diethylamide (LSD), mescaline, 2,5-dimethoxy-4-iodoamphetamine (DOI) and the agonistic effects that they have on this serotonin receptor subtype (131–133). Induction of schizophrenic symptoms performed in preclinic and clinical studies have demonstrated the modulation of the glutamate transmitter system *via* the use of ketamine and phencyclidine, N-methyl-D-aspartate (NMDA) receptor antagonists (130). With a new class of mGlu_2/3_ agonists emerging as potential antipsychotic drugs, LY378268 and LY404039 (134), the known functional antagonism between both mGlu_2_ and 5-HT_2A_ receptors meant that investigations could be carried out on whether a direct interaction between these two receptors could occur. Studies demonstrate that coupling of G_i/o_ proteins to the mGlu_2_ protomer that is distal from the 5-HT_2A_ receptor, is necessary for mGlu_2_ to perform cross-talking with 5-HT_2A_ in order for activation of G_q/11_ signalling and calcium releasing to occur (103). These findings are crucial in order to understand the pharmacology that occurs between such receptors so that the design and synthesis of ligands that target these heterodimeric complexes can be successful in tackling disorders such as schizophrenia. Peptides could be of significance as they have the advantage of being able to target the PPIs and cover the large interfaces that are involved, whilst also having potential therapeutic benefits and decreased likelihood of off-target side effects.

### Peptides Approved as Therapeutics and in the Pipeline

The majority of peptides that target class A GPCRs are receptor agonists and are used to either improve the function of the endogenous peptide, or substitute it with their high potency and affinity (EC_50_ = 8.5; K_i_ = 8.4, respectively) (32). Although they possess a higher potency and efficiency than most clinically approved drugs, the short plasma half-life can become a huge drawback for peptides as they are rapidly excreted and degraded by peptidase enzymes and levels of distribution are low due to their hydrophilic and polar characteristics (32). As a result, only certain clinical indications can really benefit from these low half-life drugs being administered. There are currently 26 peptides that have been synthesized for therapeutic purposes, with the majority having an agonistic effect (135). There are a number of peptides that are currently in clinical trials that could hope to provide some promising results if they are brought forward to clinic, including peptide antagonists as a potential anticancer agent for the class A GPCR, CXCR4 (136). As previously mentioned there is, an approved selective agonist for the MC4, setmelanotide; it is a synthetic peptide that is used to treat obesity (55). There are several peptides in clinical trial stages targeting GPCRs as well as approved therapeutics, which highlight not only the importance of peptides as drugs, but also the relevance and potential success they can lead to in clinic.

## Concluding Remarks

PPIs are arguably one of the most complex interactions to target therapeutically. The size and nature of these interactions has raised a multitude of challenges of how these surfaces can be targeted. Over the past decade, there have been some crucial discoveries in this field, mainly due to the increasing understanding of the nature of PPIs and ways in which they can be targeted. Due to the discovery of GPCRs existing as oligomeric complexes, the detection of PPIs and therefore the necessity for drugs that target these interactions is necessary. In this review, we have demonstrated that the use of peptides as drugs is an important method to target PPIs for GPCRs. From being deemed ‘undruggable’ to having peptides in the pipeline and in clinic, the knowledge about PPIs and peptides has come a long distance. Improving this field of drug design and development can be done by providing better mapping of these interactions, the expansion of structural information of GPCR oligomeric complexes, development of *in vivo* techniques to aid the localisation of the dimers, as well as methods in refining peptides, such as optimisation of stapled peptides. These are some of the ongoing challenges that we are faced with, however with the developing technologies and strategies approaching these matters, and the pioneering work that has already been performed and discovered, the future is hopeful for the use of peptides as therapeutics agents for targeting GPCR PPIs.

## Author Contributions

LAH and PJM initiated the manuscript. ZF provided the first draft of the manuscript and generated the figures. All authors contributed to the article and approved the submitted version.

## Funding

This work was supported by BBSRC grants BB/R0006946/1 and BB/V00719X/1 and Barts Charity award MRC0227 to PM.

## Conflict of Interest

The authors declare that the research was conducted in the absence of any commercial or financial relationships that could be construed as a potential conflict of interest.

## Publisher’s Note

All claims expressed in this article are solely those of the authors and do not necessarily represent those of their affiliated organizations, or those of the publisher, the editors and the reviewers. Any product that may be evaluated in this article, or claim that may be made by its manufacturer, is not guaranteed or endorsed by the publisher.
